# Childhood Blindness: A Rare Case of Leber Hereditary Optic Neuropathy in a 16-Year-Old Egyptian Patient

**DOI:** 10.7759/cureus.71619

**Published:** 2024-10-16

**Authors:** Abdulla I Abuhamaid, Ghufran S Alsaffar, Zainab H Madan, Yahya Wahba

**Affiliations:** 1 Department of Pediatrics, Faculty of Medicine, Mansoura University, Mansoura, EGY

**Keywords:** childhood blindness, incomplete penetrance, leber hereditary optic neuropathy, lhon, maternal inheritance, mitochondrial dna mutation, mtdna

## Abstract

Leber hereditary optic neuropathy (LHON) is a maternally inherited mitochondrial genetic disorder that is rarely encountered in daily clinical practice. It presents by an acute or subacute onset and a progressive course of painless, bilateral, sequential severe loss of vision, mostly seen in young males. Mutations in the mitochondrial DNA in these patients lead to dysfunction at complex I of the respiratory chain, causing a selective degeneration of the retinal ganglion cells and predisposition toward the development of the clinical symptoms. LHON can mimic other ophthalmological disorders, making it a commonly misdiagnosed and mismanaged disease. Management of LHON remains mainly supportive, as a definitive cure for this condition is yet to be developed. In this case report we describe the disease progression encountered in a 16-year-old Egyptian male patient diagnosed with LHON, highlight the difficulties encountered in diagnosing and managing this case, and review the literature on LHON.

## Introduction

Leber hereditary optic neuropathy (LHON) is a rare, maternally inherited mitochondrial genetic disorder characterized by the acute or subacute onset of painless bilateral loss of vision that progresses over weeks to months primarily affecting young males. The disorder was named after the German ophthalmologist Theodor Leber who described its clinical characteristics back in 1871. Ninety-five percent of LHON cases are caused by any of the three major mutations in mitochondrial DNA (mtDNA) m.11778G>A, m.14484T>C, and m.3460G>A. These mutations lead to abnormalities in the ND1, ND4, and ND6 subunits of complex I of the respiratory chain, leading to defective respiratory chain function and excess radical oxygen species (ROS) production. This disruption appears to selectively affect the retinal ganglion cells (RGCs), leading to their damage and eventual atrophy which explains the visual disturbance encountered in this condition [1]. Due to its rarity in clinical practice, this disorder is commonly overlooked, mismanaged, and only diagnosed at late stages when the beneficial therapeutic window is already missed.

Here, we report a case of LHON leading to visual loss in a 16-year-old Egyptian male patient, highlighting difficulties encountered during the diagnosis and management of this case, and providing an up-to-date clinical discussion to increase the awareness about this disorder.

## Case presentation

**Figure 1:**
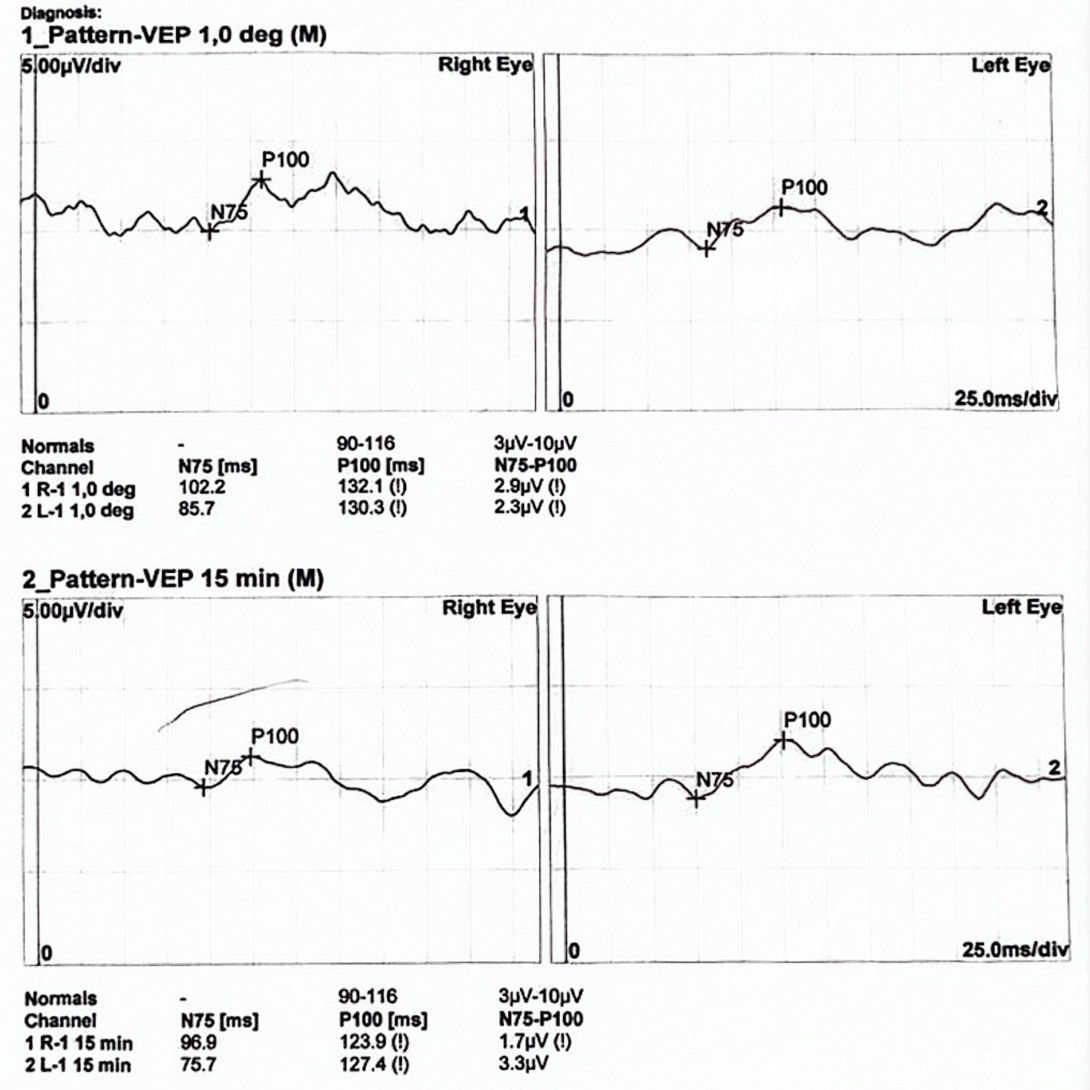
VEP results from July 2023. VEP of the right eye revealed poorly formed responses with a marked delay in retinocortical transmission time (when stimulated with a large check size) and a moderately delayed retinocortical transmission time (when stimulated with a small check size) with overall marked reduced amplitude. VEP of the left eye revealed moderately formed responses with a marked delay in retinocortical transmission time (when stimulated with a large check size) and a moderately delayed retinocortical transmission time (when stimulated with a small check size) with overall moderately reduced amplitude. VEP, visual evoked potential.

**Figure 2:**
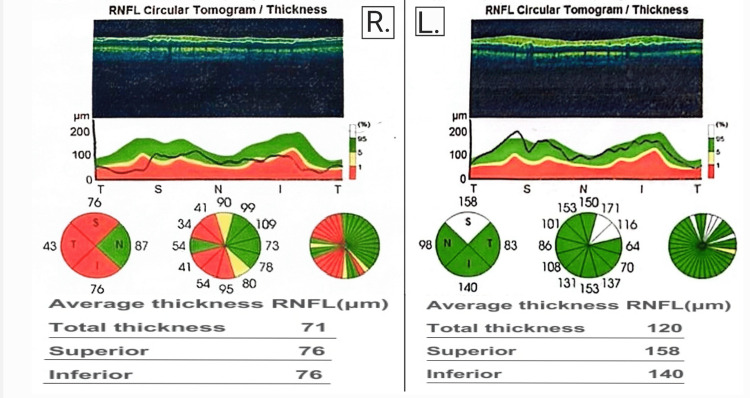
OCT results from July 2023. OCT reveals thinning of the RNFL in all quadrants except the nasal quadrant in the right eye (R), and an increase in the thickness of the RNFL in the nasal quadrant in the left eye (L). OCT, optical coherence tomography; RNFL, retinal nerve fiber layer.

A 16-year-old Egyptian male patient presents with a sub-acute onset of bilateral progressive painless diminution of vision that developed over 15 months. Initially in June 2023, the patient’s parents noticed a squint in his right eye following a three-day-period febrile illness, a few days later the patient subjectively felt deterioration in the vision in his right eye which led him to seek medical attention. Detailed historical inquiry revealed that the patient was exposed to black smoke during a camping trip and developed fever, eye strain, and a squint in his right eye with no other associated symptoms. The patient’s medical records revealed no systemic or congenital diseases, no drug or tobacco use, and no family history of ophthalmological disorders or consanguinity. His surgical history was free. His vitals were stable and general examination revealed no abnormalities. Visual acuity testing revealed an acuity of 6/9 for the right eye and 6/6 for the left eye. All other ophthalmological and laboratory tests were within normal ranges. A few weeks later in July of the same year, the patient presented to the hospital complaining of slight deterioration in the visual acuity of his left eye. A pathology in the optic nerves or brain was suspected. Thus, the patient was scheduled to undergo brain magnetic resonance imaging (MRI), visual evoked potentials (VEP) testing, and optical coherence tomography (OCT). MRI revealed an increased short T1 inversion recovery signal of the right optic nerve suggesting right-sided optic neuritis. VEP revealed marked affection of conduction on the right side and moderate affection on the left side (Figure 1). OCT revealed left optic nerve head swelling and right diffuse peripapillary RNFL (retinal nerve fiber layer) thinning identified as post-swelling atrophy of the RNFL (Figure 2). The patient was admitted to the hospital and received intravenous methylprednisolone for five days to improve the visual deterioration, but no improvement was noticed. He was discharged and scheduled for follow-up in the neuro-ophthalmology clinic. Differential diagnoses at that point were Devic’s disease, clinically isolated demyelinating syndrome, and toxic optic neuritis. Laboratory and radiological investigations for systemic lupus, vasculitis, demyelinating diseases, and metabolic diseases all came back negative.

**Figure 3:**
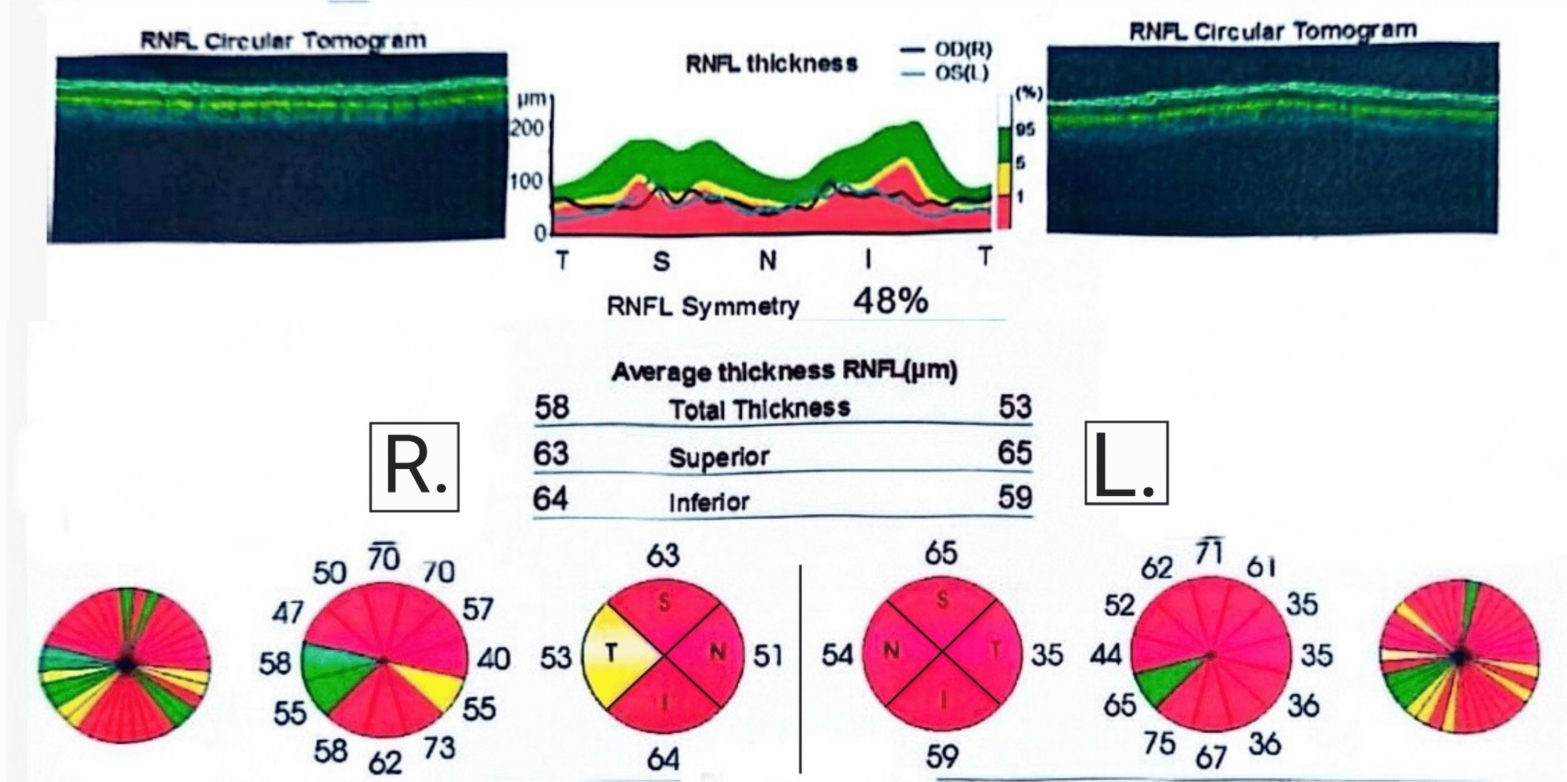
OCT results from April 2024. Advanced damage to the RNFL in both eyes has been revealed. OCT, optical coherence tomography; RNFL, retinal nerve fiber layer.

The patient was kept on oral steroids for the following eight months. Despite that, his visual acuity continued to deteriorate reaching counting fingers (CF) in both eyes by the end of March 2024. The continuous visual deterioration, the sequence of disease progression, the patient’s gender and age, and the unresponsiveness to the given therapy introduced the possibility that the patient was suffering from a rare disease caused by a mitochondrial genetic aberration that affects males at this age group called LHON. The patient was re-admitted to the hospital in April 2024 and was thoroughly examined revealing no abnormalities other than visual acuity of CF in both eyes. OCT was done again to monitor the disease progression and revealed bilateral advanced damage to the optic discs and RNFL (Figure 3). The patient was started on mitochondrial cocktail supplements (composed of carnitine, vitamin C, coenzyme Q10, Epicozym, and biotin) and was instructed to undergo genetic testing for mtDNA mutations which revealed a point mutation at the MT-ND4 gene, confirming the diagnosis of LHON (Table 1). Thus, visual rehabilitation and visual aids were provided for the patient, and genetic counseling was started for his family members.

**Table 1:** Genetic testing results. The m.11778G>A p.(Arg340His) variant in the MT-ND4 gene was detected in a homoplasmic state (100% of 4123 NGS "Next Generation Sequencing" reads), confirming LHON diagnosis. LHON, Leber hereditary optic neuropathy.

Gene	Variant Coordinates	Amino Acid Change	Zygosity	Type and Classification
MT-ND4	NC_012920.1:m.11778G>A	YP_003024035.1:p.Arg340His	Homoplasmic (100% of 4123 NGS reads)	Missense pathogenic (class 1)

**Figure 4:**
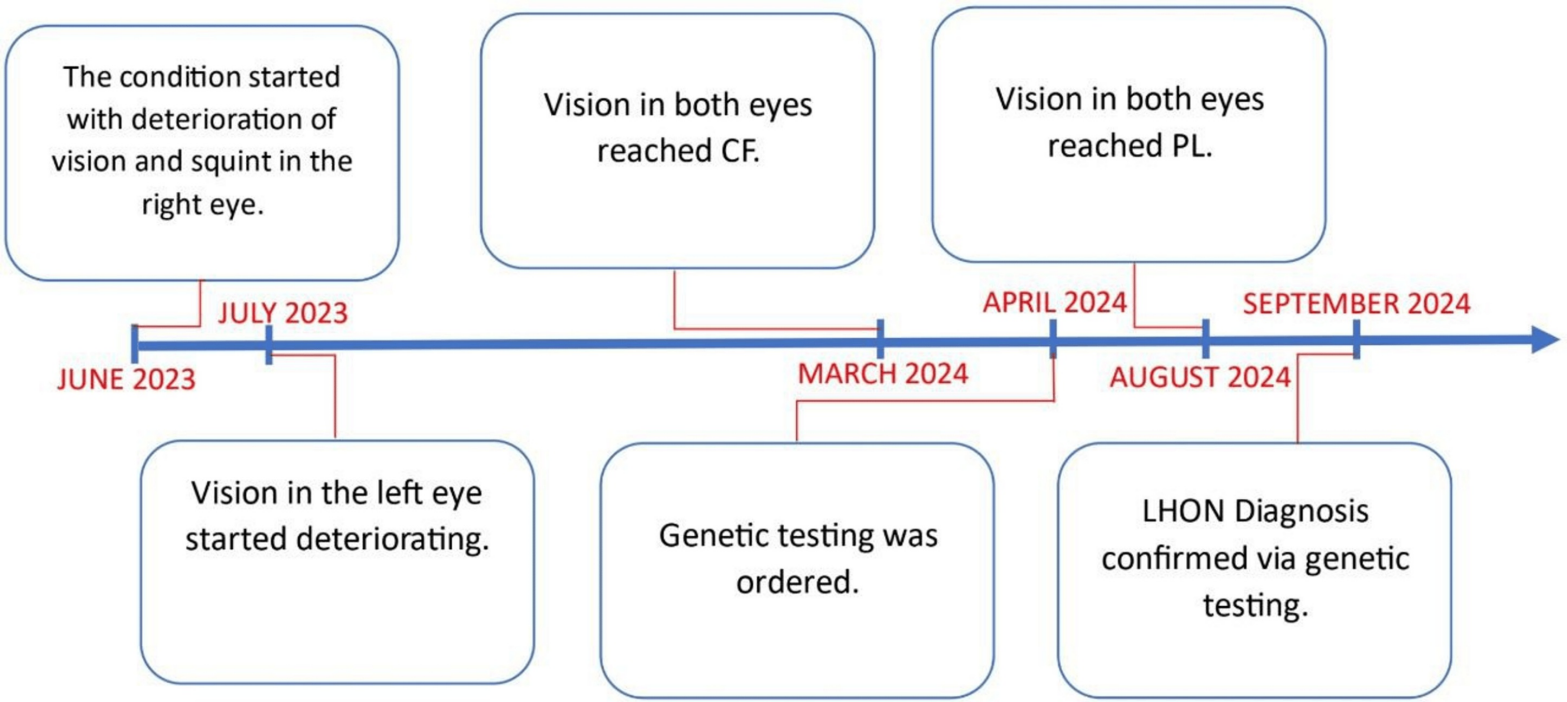
Summarized timeline of the progression of the disease encountered in this case. CF, counting fingers; PL, perception of light; LHON, Leber hereditary optic neuropathy.

On follow-up in August 2024, an examination revealed that the vision deteriorated further and now the visual acuity in both eyes stands at perception of light. Fundoscopy revealed optic atrophy on both sides. Other neurological and systematic examinations were normal. Another OCT was done for the patient and revealed bilateral thinning and atrophy of both the ganglion cell layer (GCL) and RNFL. A summarized timeline of the progression of the condition in our patient is shown in Figure 4.

## Discussion

LHON is characterized by an acute or subacute onset of painless bilateral, simultaneous, or sequential loss of central vision primarily affecting young males. Even though LHON is a rare clinical entity, it is still the most common inherited mitochondrial disorder, having an estimated worldwide prevalence of 1 in 50,000, with some variability across different worldwide regions [1]. Classically, LHON has been widely regarded as a disease of young males peaking at ages 14-26 years, with a male-to-female ratio of 5:1. However, a recent international study conducted by Poincenot et al. suggests that the ratio is closer to 3:1. Interestingly, at ages younger than five and older than 45, the male-to-female ratio becomes almost 1:1. Moreover, although the disease is more prevalent in young adults, it can manifest at any age [2]. m.11778/ND4 is the most prevalent mutation accounting for almost 70% of cases and having the worst prognosis regarding visual recovery. While the two other primary mutations have almost similar prevalences, m.14484/ND6 has by far the best visual prognosis. In most LHON cases, the primary mutation is homoplasmic (meaning the mutation is present in all the maternally inherited mtDNA). However, heteroplasmy (meaning the presence of a mix of mutated and wild-type mtDNA) is found in 10-15% of LHON cases. In such cases, the phenotypic expression of the disease appears to be correlated with the mutation load as the risk of disease expression falls significantly if the mutation load is less than 60% [3].

An interesting fact about LHON genetics is that the previously mentioned mutations have incomplete penetrance, which means that the presence of these mutations does not guarantee the development of LHON. Sex is an important modifier for the phenotypic expression of LHON. Studies reveal that 50% of males with an underlying LHON mutation develop vision loss, but only 10% of females with an underlying LHON mutation go on to develop vision loss. This difference is attributed to the protective effects of estrogen which increases mitochondrial biogenesis, decreases ROS production, and decreases RGC apoptosis. Moreover, recent studies on some X-linked modifier genes such as PRICKLE3 on animal subjects revealed that PRICKLE3-deficient mutants have a higher risk of expressing LHON. Also, environmental risk factors such as smoking, alcoholism, exposure to chemical toxins, use of antiretroviral or antituberculosis drugs, and exposure to stressful events like undergoing major surgeries have been linked to an increased risk of LHON disease expression [3]. This might explain why our patient had preserved vision for 16 years, up until he was exposed to environmental hazards, which in combination with his febrile illness led to activation of the disease expression.

Clinically, LHON is classified into a presymptomatic phase, an acute phase, and a chronic phase, with each phase varying in duration between different patients [3].

In the presymptomatic phase, the patient does not complain of any visual symptoms. However, ophthalmological examination may reveal signs such as peripapillary telangiectasias and disc pseudo-edema (defined as swelling of the RNFL without leakage of dye on fluorescein angiography). OCT at this stage may reveal dynamic thickening of the RNFL mostly at the temporal and inferior quadrants with no abnormalities in the GCL [3,4].

As for the acute phase, patients complain of acute deterioration in visual acuity, visual field defects in the form of central or centrocecal scotomas, and color vision abnormalities. These symptoms usually start in one eye with eventual involvement of the fellow eye within weeks to months. Ophthalmological evaluation in this phase may reveal pseudo-edema, vascular telangiectasias, and optic disc hyperemia. But, in 20-40% of LHON patients, fundoscopy comes back negative, further increasing the difficulty of suspecting LHON [3]. In the instance of our case, the patient expressed the typical symptomatic progression of LHON, but his fundoscopic examination revealed no abnormalities which led us to falsely focus our efforts on other etiologies resulting in a delay in diagnosis. OCT at this stage reveals a reduction in the thickness of the GCL and RNFL [4]. VEP can be useful and can reveal abnormalities in signal conduction [5]. Moreover, MRI is used to exclude other abnormalities like multiple sclerosis and can demonstrate optic nerve enhancement in post-contrast images in the acute phase of LHON [1,3].

The chronic phase is known via the development of optic atrophy. At this point, the visual deterioration reaches a plateau and the chances of recovering better vision become very minimal [3]. OCT at this stage reveals significant thinning in both the GCL and RNFL [4,6]. On rare occasions, individuals with LHON might have extra-ocular manifestations, and such cases are termed LHON Plus syndromes; they might complain of cardiac arrhythmias, peripheral neurological manifestations, ataxias, myopathies, movement disorders, and neuropsychiatric disorders, and some cases might even develop features of multiple sclerosis termed Harding’s disease [3].

Diagnosing LHON is a challenging task as it requires the exclusion of all other etiologies for such manifestations, such as demyelinating, compressive, nutritional, or toxic optic neuropathies. Investigations for LHON diagnosis include visual field perimetry, visual acuity testing, fundus examination, color vision testing, and OCT. Additional tests like VEP, MRI, and electroretinograms can also be used to gain additional clinical data and exclude other disorders. To confirm the diagnosis, genetic molecular testing for mtDNA mutations is required [1,3,7].

**Figure 5:**
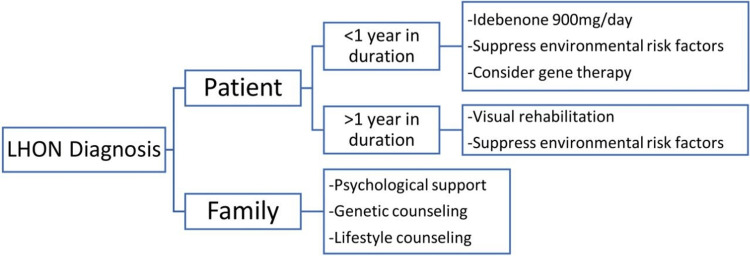
Algorithm for the management of LHON. This algorithm was suggested by Hage and Vignal-Clermont in the article (Hage R, Vignal-Clermont C: Leber hereditary optic neuropathy: review of treatment and management. Front Neurol. 2021:651639) [11]. The use, reproduction, and distribution of the data provided in the figure was permitted by the original publisher. LHON, Leber hereditary optic neuropathy.

There is no known cure for LHON. The aims of management of LHON are reducing time for visual recovery, providing supportive care for the patient and their family, decreasing the oxidative stress caused by excess ROS, avoiding environmental agents that have a deleterious effect on mitochondrial activity, providing visual rehabilitation and aids for patients with intact peripheral vision, providing constant follow-ups to monitor the disease progression, and providing genetic counseling for the patient and their relatives [3,8]. Coenzyme Q10, vitamin B12, lutein, and other over-the-counter supplements are widely used due to their minimal risk profile and theoretical benefits in lowering oxidative stress. Unfortunately, compelling evidence supporting the use of these agents is lacking [9]. A synthetic coenzyme Q10 analog called idebenone has been studied thoroughly and was found to have a beneficial effect in promoting visual recovery in LHON especially when used in the early stages of the disease; it functions as an electron carrier that bypasses the defect in complex I. It is currently approved by the European Medicines Agency (EMA) and awaiting the Food and Drug Administration (FDA) approval. Another ubiquinone analog that has superior efficacy than idebenone called EPI-743 is currently in the experimental phase [3,8,10]. Finally, gene therapy for m.11778/ND4 mutations is currently under study and is showing promising results. Gene therapy simply aims to replace the mutated gene with a normal gene carried by an innate vector into the patient’s cells [8]. An algorithm proposed by Hage and Vignal-Clermont for the management of LHON is shown in Figure 5 [11].

## Conclusions

LHON is a rare mitochondrial genetic disease more commonly encountered in young males, characterized by the sudden onset of visual deterioration starting in one eye and followed by the other eye within weeks or months. LHON mutations have incomplete penetrance, meaning that not all people who harbor those mutations are guaranteed to express the disease. Many risk factors have been implicated in increasing the risk of disease expression like being male, exposure to environmental toxins, smoking, alcohol abuse, and some drugs. Clinically, LHON mimics other ophthalmological disorders, making it notoriously difficult to diagnose requiring a high degree of clinical suspicion and experience. Unfortunately, there is still no known cure for this condition, but the introduction of ubiquinone analogs like idebenone and the advances witnessed in gene therapy are very promising. Our take-home message is to encourage healthcare providers to add LHON in their differential diagnoses in cases presenting with acute bilateral visual loss, especially in young males, even in the absence of fundoscopic signs or a positive family history.
